# Duodenal Mucinous Adenocarcinoma in a Patient With Immunodeficiency: A Case Report

**DOI:** 10.7759/cureus.34509

**Published:** 2023-02-01

**Authors:** Timon Sseruwagi, Joel Musinzi, William M Mutumba, Catherine Lewis

**Affiliations:** 1 General Surgery, Kampala International University, Ishaka, UGA; 2 Internal Medicine, St. Joseph's Hospital Kitovu, Masaka, UGA; 3 Anatomical Pathology, Lancet Laboratories, Kampala, UGA; 4 General Surgery, East Tennessee State University, Johnson City, USA; 5 General Surgery, St. Joseph's Hospital Kitovu, Masaka, UGA

**Keywords:** mucin, adenocarcinoma, duodenum, immunodeficiency, gastrointestinal bleeding, esophagogastroduodenoscopy

## Abstract

Duodenal mucinous adenocarcinoma is a rare type of small bowel carcinoma. It is not commonly encountered; hence little knowledge exists about its presentation, diagnosis, and management. The diagnosis is mostly made by either esophagogastroduodenoscopy (EGD) or intra-operatively. Some of the main symptoms are abdominal pain, nausea, vomiting, weight loss, or signs and symptoms of upper gastrointestinal bleeding. Therefore, this is a serious condition that healthcare providers and patients should be aware of to reduce its severity and improve prognosis. We present a case of duodenal mucinous adenocarcinoma in a patient with immunodeficiency virus.

## Introduction

The small bowel contains up to 3% of all cancers of the gastrointestinal (GI) tract, with 25-40% being adenocarcinomas [1,2]. The incidence of adenocarcinoma of the small bowel is highest in the duodenum (up to 55%). However, duodenal adenocarcinoma (DA) makes up about 0.3% of all GI tract malignancies [1-4]. Hamburger was the first to describe DA in 1746. It is more common in males aged 40-70 and is more commonly located in the second portion of the duodenum [5,6]. DA is among the most common causes of death in patients with small bowel malignancies, and patients typically present at a late stage. Predisposing factors for small bowel adenocarcinomas and specifically DA include familial conditions such as Lynch syndrome, familial adenomatous polyposis (FAP), hereditary nonpolyposis colorectal cancer (HNPCC), and Peutz-Jeghers syndrome (PJS) [1,4-8].

Duodenal mucinous adenocarcinoma (DMA) is an uncommon malignancy of the small bowel. DMA is a distinct pathological entity that originates from the epithelium and is characterized by increased production of mucin that makes up greater than 50% of the adenocarcinoma [1,9,10]. Only eight known cases of DMA are reported in the literature [1,3,9-14]. To the best of our knowledge, we present the first case of stage IV DMA in a patient with human immunodeficiency virus (HIV).

## Case presentation

**Figure 1:**
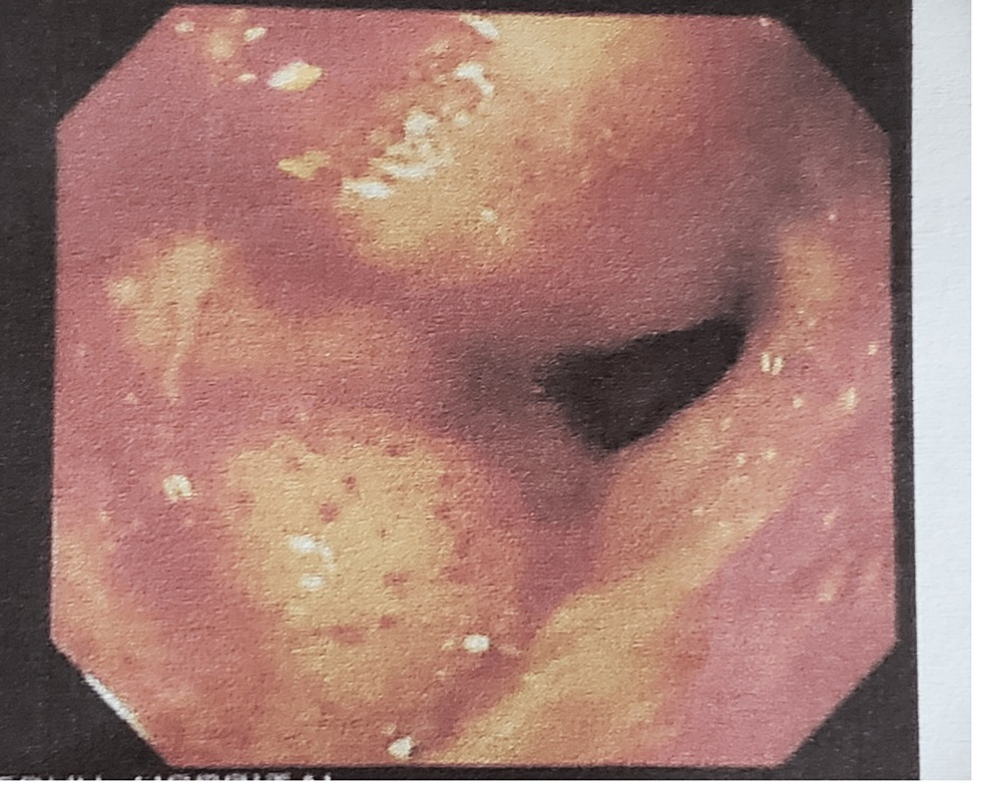
Esophagogastroduodenoscopy Bleeding and friable mass located in first portion of the duodenum with narrowing of the lumen.

A 67-year-old female presented to the outpatient clinic with a one-year history of epigastric abdominal pain that had worsened over the past one month. The pain was associated with bloating and anorexia. There were no fevers. The patient was tolerating an oral diet. Past medical history was significant for diabetes mellitus controlled with metformin and HIV controlled with highly active antiretroviral therapy (HAART). The patient was widowed and worked as a farmer. There was no history of alcohol or tobacco use. She was also being managed for chronic gastritis, and an upper endoscopy was recommended. An esophagogastroduodenoscopy (EGD) was attempted the next day. However, it was aborted due to retained food contents despite the patient fasting overnight. There was noted to be some bleeding coming from the pylorus, and the patient was started on intravenous tranexamic acid 500 mg three times daily. The patient was kept nil per os (NPO), and the EGD was repeated the following day. The EGD revealed a friable and bleeding mass in the first portion of the duodenum, from which biopsies were taken due to concerns for malignancy (Figure 1). A computed tomography (CT) scan of the abdomen was recommended to rule out metastatic disease. The patient was continued on oral omeprazole 40 mg daily and tranexamic acid and told to follow up in two weeks for pathology review.

**Figure 2:**
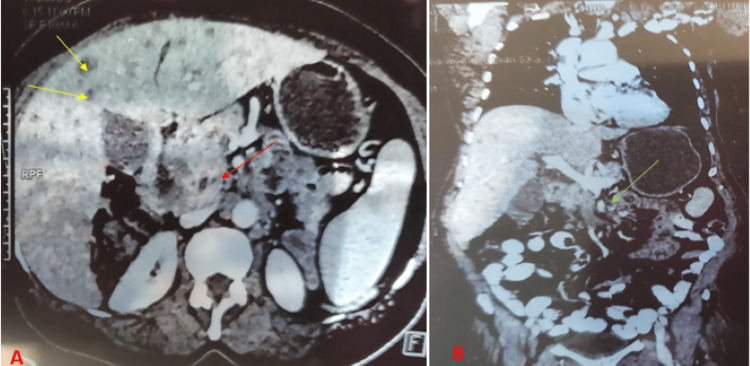
Computed tomography (CT) of the abdomen and pelvis A) Axial CT scan demonstrating duodenal mass with invasion of the pancreas (red arrow) and metastatic liver lesions (yellow arrow). B) Coronal CT image demonstrating thrombus of the superior mesenteric vein (green arrow).

**Figure 3:**
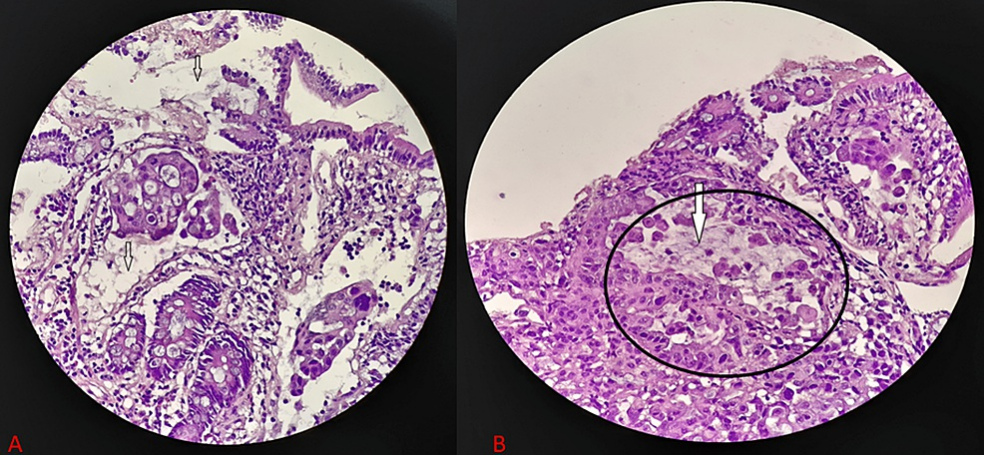
Histopathology A) Hematoxylin and eosin (H&E) staining demonstrating sheets of malignant epithelial cells forming gland-like structures. There is mucin in the stroma represented by the arrows. B) Increased magnification with areas of adenocarcinoma (black circle) with approximately 50% of mucin (arrow).

The patient returned one week later with worsening epigastric abdominal pain and decreased appetite. She had obtained a CT scan that demonstrated a large duodenal solid mass with pancreatic invasion, metastasis to the liver, intra-abdominal lymphadenopathy, and superior mesenteric vein thrombus (Figure 2). Histology results were available and demonstrated findings consistent with mucinous adenocarcinoma of the duodenum (Figure 3). The patient was subsequently admitted for further management.

On physical examination, vital signs were within normal limits. She was afebrile with mild pallor and mild jaundice. The abdomen was of normal fullness with tenderness in the epigastric region with a palpable epigastric mass. Laboratory findings were significant for a hemoglobin of 6.4 g/dL (ref. 11.5-15.1 g/dL). Sodium was decreased at 126 mmol/L (ref. 136-145 mmol/L), and chloride was 94 mmol/L (ref. 98-107 mmol/L). All other serum electrolytes and renal function tests were within normal limits. CD4 count was 363 cell/µl. Liver function tests were elevated, as shown in Table 1.

**Table 1:** Liver function tests

Lab test	Result	Reference range
Aspartate transaminase (AST)	165.7 U/L	0-40 U/L
Alkaline phosphatase (ALP)	230.9 U/L	30-120 U/L
Alanine transaminase (ALT)	94.9 U/L	0-50 U/L
Total protein	8.0 g/dL	6.6-8.7 g/dL
Direct bilirubin	5.9 mg/dL	0-0.5 mg/dL
Total bilirubin	7.0 mg/dL	0-1.2 mg/dL
Albumin	36.29 g/L	39.7-49.4 g/L
Gamma-glutamyltransferase (GGT)	356.2 U/L	5-61 U/L

**Figure 4:**
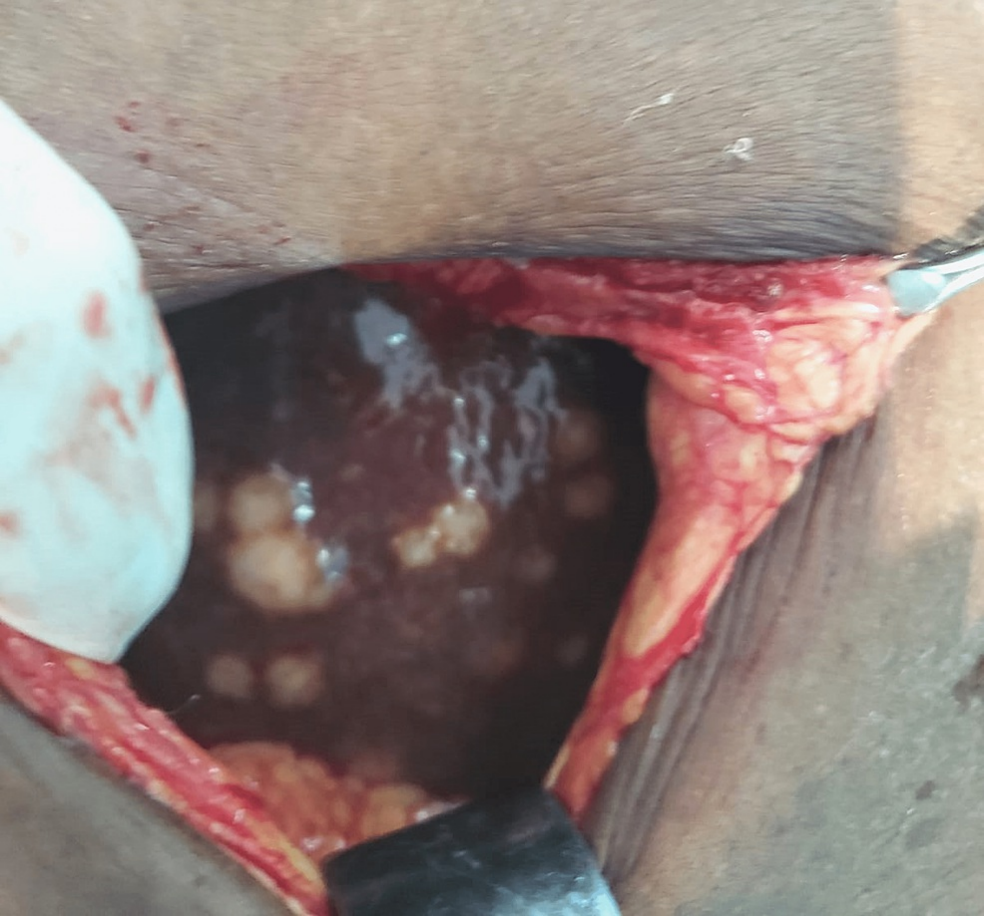
Liver metastasis Intraoperative findings of liver metastasis.

The patient was allowed to feed orally as tolerated. However, she began to develop abdominal distention and vomiting. Intravenous tranexamic acid 500 mg three times daily and pantoprazole 40 mg daily were initiated. Pain was controlled with intravenous paracetamol one gram every eight hours, and the patient was transfused with packed red blood cells for anemia. After a discussion of all treatment options, the patient agreed to a palliative procedure. A Roux-en-Y gastrojejunostomy and jejunojejunostomy were performed on the eighth day of admission. Intraoperatively, the patient was noted to have a fixed mass of the duodenum with enlarged mesenteric lymph nodes and metastasis to the liver (Figure 4). The patient had a return of bowel function on postoperative day five, and oral sips were initiated. The patient requested discharge on postoperative day seven due to financial constraints and was lost to follow-up.

## Discussion

DA comprises 0.3% of all GI tract malignancies [2,5,11]. Most cases are found in the second portion of the duodenum or are periampullary [3,6]. It more commonly affects males, and African Americans are more likely to be diagnosed with small bowel adenocarcinoma and have a higher mortality. Risk factors for DA are divided into modifiable and non-modifiable. Non-modifiable risk factors include race and ethnicity, age, sex, hereditary conditions (i.e., FAP, Lynch syndrome, PJS), inflammatory bowel disease, and celiac disease. Modifiable risk factors include a high-fat diet, alcohol consumption, tobacco use, obesity, biliary tract diseases, chronic use of non-steroidal anti-inflammatory drugs (NSAIDs) or corticosteroids, and certain occupational hazards [3, 9-12]. For the patient under discussion, there are no identifiable risk factors other than advanced age.

DA is staged according to the 2010 American Joint Committee on Cancer's (AJCC) tumor-nodal-metastasis (TNM) system [6], as shown in Table 2. The clinical manifestations of DA are nonspecific, making the diagnosis difficult. However, there is a progressive manifestation of the signs and symptoms as the condition advances toward stage IV. Patients typically present with abdominal pain, unexplained weight loss, GI bleeding, acid reflux, nausea, vomiting, and jaundice [1,3,6,10]. Early diagnosis is usually difficult to attain since the condition may be asymptomatic or have vague symptoms that overlap with other GI conditions. Therefore, patients present at a later stage [1, 3]. Metastasis is present in more than 50% of patients at the time of diagnosis [6]

**Table 2:** American Joint Committee on Cancer (AJCC) tumor-nodal-metastasis (TNM) staging for duodenal adenocarcinoma

Category and stage	Characteristic
Primary tumor (T)	
Tx	Primary tumor cannot be assessed
T0	No evidence of primary tumor
Tis	Carcinoma in situ
T1a	Tumor invades lamina propria
T1b	Tumor invades submucosa
T2	Tumor invades muscularis propria
T3	Tumor invades through the muscularis propria and into the subserosa or perimuscular tissue
T4	Tumor perforates the visceral peritoneum or directly invades other organs
Regional lymph nodes (N)	
Nx	Regional lymph nodes cannot be assessed
N0	No regional lymph node metastasis
N1	Metastasis in 1 to 3 regional lymph nodes
N2	Metastasis in 4 or more regional lymph nodes
Distant metastasis (M)	
M0	No distant metastasis
M1	Distant metastasis
Stage	
0	TisN0M0
I	T1-2 N0 M0
IIA	T3 N0 M0
IIB	T4 N0 M0
IIIA	Any T N1 M0
IIIB	Any T N2 M0
IV	Any T Any N M1

The primary differential diagnosis for DA is other periampullary tumors, such as pancreatic carcinoma or cholangiocarcinoma [6]. Radiology and endoscopy are important aspects of making the diagnosis. Endoscopy allows for simultaneous visualization and biopsy. Visualization of tumors that involve the third or fourth portions of the duodenum is difficult and may be missed, making longer fiberoptic scopes useful. Endoscopic ultrasound may allow for the evaluation of associated lymphadenopathy [15]. Mucin may also be visualized on endoscopy [9, 11]. CT is the most commonly used imaging modality. CT findings demonstrate a concentric or irregular thickening of the bowel wall. There may also be a fungating mass with or without the narrowing of the lumen. CT findings in T4 tumors demonstrate direct invasion of surrounding structures and/or obliteration of the fat plane between the duodenum and adjacent structures, as in our patient. MRI is an adjunct that helps to delineate the relationship of the duodenal tumor to adjacent structures [6]. On histological examination, DMA is characterized by mucin that encompasses greater than 50% of the adenocarcinoma, as demonstrated in Figure 3 [1, 9, 10]. CDX2, a marker for colorectal cancer, has been expressed in DA [15]. Due to the rarity of DMA, the exact immunohistochemical profile is not known.

The most effective and suggested treatment is surgical resection. In tumors involving the first and second portions of the duodenum, pancreaticoduodenectomy is required. Adequate lymphadenectomy is imperative to improve prognosis [6, 15, 16]. Even with complete surgical resection, a complete cure is only achievable in up to 25% of patients [2]. Adjunct chemotherapy may be recommended in those with lymph node-positive disease or those with distant metastasis. However, the exact chemotherapy regimen has not been standardized [2, 6, 15]. Neoadjuvant chemotherapy should be offered on a case-by-case basis [5, 6]. Palliative chemotherapy may be considered in those who have unresectable disease [17]. Palliative resection may be necessary for unresectable disease in those with uncontrolled bleeding, gastric outlet obstruction, biliary tract obstruction, or for pain relief [6, 15]. Our patient had metastatic disease with gastric outlet obstruction and bleeding. Therefore, a palliative gastrojejunostomy was performed.

Human immunodeficiency virus (HIV) and acquired immunodeficiency syndrome (AIDS) have been associated with various malignancies. The GI tract is a major site of disease in immunodeficiency patients. Dyspepsia is a common symptom of HAART [18] and was present in our patient. Kaposi sarcoma and non-Hodgkin lymphoma (NHL) are the most common malignancies diagnosed and frequently involve the GI tract [19, 20]. The majority of NHLs in the GI tract are B-cell lymphomas, which can be difficult to differentiate from adenocarcinoma. Both can present as ulcerated lesions or masses, in which endoscopy and biopsy facilitate the diagnosis [19]. Other than anorectal carcinoma, epithelial lesions of the GI tract in immunodeficiency patients are rare [21]. To the best of our knowledge, we present the first case of a patient with both immunodeficiency and DMA.

## Conclusions

Duodenal adenocarcinoma is a rare condition while duodenal mucinous adenocarcinoma is even more rare. Its diagnosis in an immunocompromised patient has not been previously reported. The clinical presentation is non-specific, leading to late diagnosis, poor prognosis, and increased mortality. Prompt and proper staging is imperative for adequate management and improved survival.
